# RBP–RNA interactions in the control of autoimmunity and autoinflammation

**DOI:** 10.1038/s41422-022-00752-5

**Published:** 2023-01-05

**Authors:** Juan Liu, Xuetao Cao

**Affiliations:** 1 National Key Laboratory of Medical Immunology, Institute of Immunology, Second Military Medical University, Shanghai, China; 2 Department of Immunology, Institute of Basic Medical Sciences, Chinese Academy of Medical Sciences, Beijing, China; 3 Frontier Research Center for Cell Response, Institute of Immunology, College of Life Sciences, Nankai University, Tianjin, China

**Keywords:** Autoimmunity, RNA metabolism

## Abstract

Autoimmunity and autoinflammation arise from aberrant immunological and inflammatory responses toward self-components, contributing to various autoimmune diseases and autoinflammatory diseases. RNA-binding proteins (RBPs) are essential for immune cell development and function, mainly via exerting post-transcriptional regulation of RNA metabolism and function. Functional dysregulation of RBPs and abnormities in RNA metabolism are closely associated with multiple autoimmune or autoinflammatory disorders. Distinct RBPs play critical roles in aberrant autoreactive inflammatory responses via orchestrating a complex regulatory network consisting of DNAs, RNAs and proteins within immune cells. In-depth characterizations of RBP–RNA interactomes during autoimmunity and autoinflammation will lead to a better understanding of autoimmune pathogenesis and facilitate the development of effective therapeutic strategies. In this review, we summarize and discuss the functions of RBP–RNA interactions in controlling aberrant autoimmune inflammation and their potential as biomarkers and therapeutic targets.

## Introduction

The immune tolerance toward self-components is critical for the maintenance of immune homeostasis and prevention of the unwanted autoimmune pathology. Immune tolerance can be divided into two categories, central tolerance and peripheral tolerance. Central tolerance is the first layer of protective tolerance toward self-antigens by clonal deletion of autoreactive T cells in the thymus and by anergy, receptor editing, and clonal deletion of autoreactive B cells in the bone marrow. As a considerable proportion of T and B cells can escape from central tolerance, a second layer of peripheral tolerance is critically important to eliminate or inactivate these escaped autoreactive lymphocytes.^1^ The major mechanisms for peripheral tolerance include expression of immune inhibitory molecules, T cell anergy, B cell anergy, ignorance of specific self-antigens in immunologically privileged tissues, and generation of immune regulatory cell types such as regulatory T (Treg) cells.^2–5^ Among immune inhibitory molecules are well-known immune checkpoint molecules cytotoxic T-lymphocyte antigen 4 (CTLA-4) and programmed cell death protein 1 (PD-1). T cell anergy is a consequence of insufficient co-stimulation signals from immature or regulatory antigen-presenting cells (APCs), whereas B cell anergy is due to sustained exposure to soluble antigens or inhibitory intracellular signaling.^6^ Failure of either central or peripheral immune tolerance can elicit harmful immune responses against self-components, thus skewing immune homeostasis towards autoimmunity and autoinflammation.

Although autoimmunity and autoinflammation are traditionally characterized as auto-reactive adaptive immunity and hyperactivation of innate immunity, respectively, they exert overlapping effects in the induction and persistence of inflammatory pathogenesis. A mixed spectrum of autoimmunity and autoinflammation has been identified in distinct autoimmune diseases (AIDs) and autoinflammatory diseases, as well as a series of inflammatory disorders associated with sterile inflammation.^7^ AIDs are caused by pathogenic autoimmunity and harmful inflammatory responses toward self-components. Generally, AIDs can be categorized into systemic (non-organ-specific) disorders, such as rheumatoid arthritis (RA) and systemic lupus erythematosus (SLE), and organ-specific diseases, such as multiple sclerosis (MS) and type I diabetes (T1D).^8,9^ Autoinflammatory diseases are characterized by overactivation of the innate immune system caused by monogenic mutations, such as cryopyrin-associated periodic syndromes (CAPS) linked with mutation of NOD-like receptor family pyrin domain containing 3 (*NLRP3*).^10,11^ In addition, sterile inflammation induced by non-infectious stimuli is closely related with various chronic inflammatory disorders such as atherosclerosis and neurodegenerative diseases. In general, these inflammatory disorders are associated with complex interactions among genetic, immunological, and environmental factors. The considerable heterogenicity and diversity in the underlying immunological mechanisms greatly challenge the development of effective prevention and treatment strategies against AIDs and autoinflammatory diseases. Characterizing the factors that lead to the disruption of immune tolerance and continuous inflammatory attack is therefore critical for a better understanding of autoimmune pathophysiology and the identification of clinically meaningful diagnosis markers or drug targets.

RNA–protein interactions are essential for a wide range of cellular processes related to immunity and homeostasis. RNA-binding proteins (RBPs) are fundamental mediators and regulators of RNA–protein interactions, and control various genetic, epigenetic and metabolic events in immune and non-immune cells.^12^ Canonical RBPs have RNA-binding domains (RBDs) that recognize and bind to specific sequence elements, such as adenine and uridine-rich elements (AREs), or structures, such as stem loops, of target mRNAs. A large number of non-canonical RBPs without known RBDs have also been shown to interact with RNAs and regulate immunological processes.^13,14^ For example, numerous metabolic enzymes can bind to RNA and regulate RNA function.^15,16^ Upon binding to RNAs, RBPs regulate RNA metabolism and stability mainly via post-transcriptional or translational mechanisms. RBPs can also interact with chromatin to regulate gene expression at the transcription level.^17^ In addition, RBPs have broad roles in modulating protein expression, localization, modifications, and activities via direct or indirect protein interactions. Thus, RBPs regulate a diverse range of cellular processes for a fine-tuned coordination of the transcriptional, translational and post-translational regulation of immune responses under both homeostatic and inflammatory conditions.

**Fig. 1:**
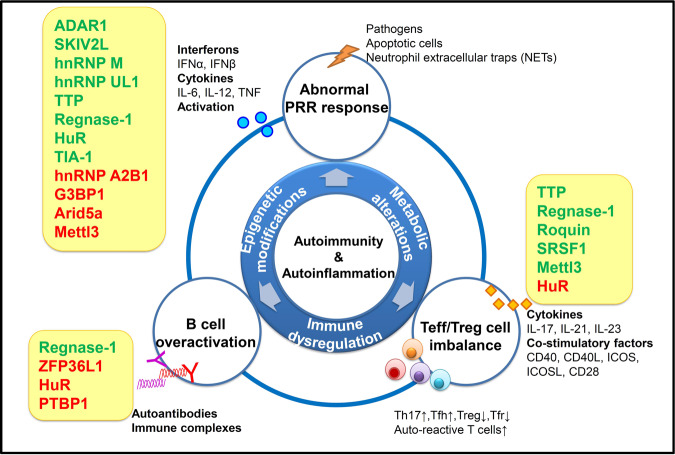
RBP-mediated regulation of immune responses in autoimmunity and autoinflammation. Immunity and inflammation are tightly controlled by regulatory networks, including epigenetic, metabolic and immunological factors. Abnormal activation of PRRs-triggered innate immunity leads to aberrant production of proinflammatory cytokines and type I IFNs, and activation of innate immune cells. The subsequent dysregulations of T cell- and B cell-dependent adaptive immunity play important roles in the breakdown of self-tolerance and the development of autoimmune pathology. RBPs are critical for mediating multi-level regulation of immune responses during autoimmunity and autoinflammation. RBPs are shown in yellow boxes next to immune responses that they target. RBPs that activate inflammatory responses or promote autoimmune pathogenesis are shown in red and those that inhibit inflammatory responses or limit autoimmune pathogenesis are shown in green. ADAR1 adenosine deaminase acting on RNA 1, SKIV2L superkiller viralicidic activity 2-like, hnRNP M heterogeneous nuclear ribonucleoprotein M, hnRNP UL1 heterogeneous nuclear ribonucleoprotein UL1, TTP tristetraprolin, HuR human antigen R, TIA-1 T-cell restricted intracellular antigen-1, hnRNP A2B1 heterogeneous nuclear ribonucleoprotein A2B1, G3BP1 GTPase-activating protein SH3 domain-binding protein 1, Arid5a AT-rich interactive domain-containing protein 5a, Mettl3 methyltransferase like 3, SRSF1 serine and arginine-rich splicing factor 1, ZFP36L1 zinc finger protein 36, C3H type-like 1, PTBP1 polypyrimidine tract binding protein 1, PRRs pattern recognition receptors, IFN interferon, IL interleukin, TNF tumor necrosis factor, Teff effector T cells, Treg regulatory T cells, Tfh follicular T helper cells, Tfr follicular regulatory T cells.

Notably, RBPs are implicated in the autoimmune pathogenesis and organ inflammation through controlling various immunological processes (Fig. 1). RBPs themselves are regulated by pathogenic and inflammatory cues through various mechanisms, such as post-transcriptional regulation and post-translational modifications. This reciprocal regulation ensures precisely controlled responses of immune cells in a context-dependent manner, contributing to a delicate balance between tolerance and immunity.^18^ In this review, we will discuss how distinct aspects of RNA metabolism, function and fate are coordinately regulated by RBP systems to control autoimmunity and autoinflammation.

## RBP–RNA interactions during autoimmunity and autoinflammation

mRNAs undergo multiple post-transcriptional regulation events throughout its life cycle, which contribute to the transcriptomic and proteomic complexity of the immune system.^19^ During the process of mRNA generation in the nucleus, RBPs participate in controlling 5′ capping, splicing and polyadenylation processes. Properly spliced and edited mRNAs are then exported to the cytoplasm, where RBPs regulate the mRNA translation initiation or silencing or decay via decapping, deadenylation, and degradation.^20,21^ These processes take place in large mRNA–RBP complexes called messenger ribonucleoprotein (mRNP), whose biogenesis, assembly, and location are essential for the maturation and fate determination of mRNAs. In particular, the CCR4–NOT deadenylase complex mediates the deadenylation and removal of the poly A tail at the 3′ end of target mRNAs, while the decapping complexes such as DCP1 and DCP2 mediate the removal of the 5′ end cap structure of target mRNAs.^22,23^ After decapping and deadenylation, the mRNAs are subjected to subsequent 5′-3′ exonucleolytic degradation by exoribonuclease 1 (XRN1) and 3′-5′ exonucleolytic degradation by exosome.

RBP-mediated modulations of mRNA metabolism and stability are essential for the regulation of inflammation and immunity.^24^ Many RBPs have potent immune regulatory functions and can regulate editing, alternative splicing, stabilization, degradation, and translation of a wide range of immune-related mRNAs. These RBPs recognize specific structures of common or distinct mRNA targets. For example, RBPs such as TTP and HuR (human antigen R) bind ARE of mRNAs encoding cytokines, chemokines, and transcriptional factors,^25–27^ whereas several others such as Roquin, Regnase, and Arid5a (AT-rich interactive domain-containing protein 5a) recognize a stem-loop structure in the 3′ UTR of the same or different mRNAs.^28,29^ These RBPs act in a cooperative or competitive manner to control innate and adaptive immune responses involved in immunity and tolerance.

Large-scale genome-wide association studies and RNA sequencing have identified many genetic variations of RBPs associated with human autoimmune or autoinflammatory diseases. These RBP mutations are closely related with immunological abnormalities in different manners. Some of these foci are overlapped across distinct inflammatory diseases (Table 1). For example, *ADAR1* (adenosine deaminase acting on RNA 1) mutations are associated with both Aicardi-Goutières syndrome (AGS) and T1D; *SKIV2L* mutations are associated with both SLE and trichohepatoenteric syndrome; AGS-associated *SAMHD1* mutants are significantly upregulated and correlated with autoinflammation in SLE patients.^30^ In addition, disruptions of RBP expression cause diversified phenotypes of chronic inflammation and AIDs in mice, in a manner dependent on the cell type or developmental stages upon conditional gene ablation (Table 2). The most critical autoimmune pathways involve innate inflammatory responses triggered by self-antigens and subsequent activation of self-reactive adaptive immunity.^31^ During these autoimmune processes, mRNAs encoding signaling molecules, proinflammatory cytokines, epigenetic regulators, metabolic enzymes, or transcriptional factors as well as self and non-self RNAs are widely targeted and regulated by RBPs. These regulations by RBPs are sometimes via their interactions with noncoding RNAs (ncRNAs) such as microRNAs (miRNAs) and long noncoding RNAs (lncRNAs). Therefore, RBPs play essential roles in the control of autoimmunity and autoinflammation via complex interactions among epigenetic, metabolic, and immunological pathways (Fig. 1).

**Table 1:** Gene variations of RBPs in human autoimmune or autoinflammatory disorders.

Human AIDs	Genetic variations of RBPs	Potential functional relevance	Cohort details
SLE	Monoallelic frameshift or missense mutations and one 3′ UTR variant of *TREX1* gene^84^	Changes in TREX1 subcellular distribution	12 out of 417 individuals with SLE
SLE	SNP rs419788 in *SKIV2L* gene^71^	Not mentioned	314 complete SLE trios (mother, father, and affected lupus proband)
SLE	Missense variant of *TLR7* (p.Tyr264His)^234^	TLR7 gain of function causing aberrant B cell survival and lupus	A Spanish girl diagnosed with SLE at the age of 7
THES	7 nonsense or frameshift mutations in *SKIV2L* gene (e.g., c.848 G>A/p.Trp283)^239^	Premature termination codon conferring loss of function	6 individuals with THES without variation in *TTC37*
Psoriasis	SNPs of *DDX58* encoding RIG-I (rs11795343), *ILF3* encoding NF90 (rs892085) and *ZC3H12C* encoding Regnase-3 (rs4561177)^240^	Related with innate immunity	From Psoriasis/Arthritis Genetics Extension (PAGE) and the Genetic Analysis of Psoriasis Consortium (GAPC) datasets
AGS	5 *TREX1* mutations (e.g., 341 G>A/R114H)^83^	Loss of TREX1 enzyme activity	10 AGS families
AGS	9 *ADAR1* mutations (e.g., c.577 C>G/p.Pro193Ala)^64^	Changes in RNA editing	12 AGS affected individuals from 8 families
AGS	Biallelic mutations in *LSM11* (c.631 G>A/p.Gly211Ser) and *RNU7-1* (encoding components of the histone pre-mRNA processing complex)^241^	Dysregulated histone RNA processing, altered cGAS distribution and enhanced STING pathway	18 AGS patients from 11 families, negative for mutations in *AGS1*–*7*
AGS	6 mutations of *IFIH1* encoding MDA5 (e.g., c.2159 G>A/p.Arg720Gln)^242^	MDA5 gain of function associated with enhanced IFN signaling pathway	11 individuals from 8 families
T1D	SNPs of *ADAR* (e.g., rs4845625), *IFIH1* encoding MDA5 (e.g., rs77088072); 3 SNPs upstream of *OAS* genes (rs4767000, rs1034687and rs739744)^243^	Dysregulation of RNA degradation pathway associated with enhanced PAMP recognition and IFN induction	From Diabetes Virus Detection (DiViD) and the network of Pancreatic Organ Donors (nPOD)
T1D	2 SNPs of *IFIH1* encoding MDA5 (rs2111485, rs984971)^244^	Noncoding variant	12,241 cases and 14,636 controls from 2 different cohorts from the ImmunoChip platform
T1D	2 variants in *IFIH1* encoding MDA5 (rs35667974/Ile923Val, and rs35337543/IVS8 + 1)^245^	Potentially reducing MDA5 function, with protective effects on T1D risk	480 T1D patients and 480 healthy controls from Great Britain from 20 DNA pools
CAD	SNP variation in RNA helicases DHX38 (rs1050362C>A), DDX59 (rs6700559C>T), DDX5 (rs1867624T>C), RBPMS2 (rs6494488A>G)^246^	Potentially related with atherosclerosis, vascular inflammation	Meta-analysis of a total of 88,192 CAD cases and 162,544 controls
Early and preclinical AD	10 variants in *RBFOX1* encoding ataxin-2-binding RBP (e.g., rs56081887, rs34860942)^247^	Increased amyloid levels associated with AD-related proteinopathy	From 6 multicenter cohort studies of healthy older individuals
AD	SNP variations in *TARBP2* (rs784567), *RNASEN* (rs10719) encoding miRNA processing elements^248^	Dysregulation of miRNA biogenesis pathway	172 AD patients and 109 healthy controls
MS	SNP variations in *ZFP36L1* (rs2236262)^249^	Likely linked to downregulated ZFP36L1 mRNA expression	80,094 individuals of European ancestry

Abbreviations: *SLE* systemic lupus erythematosus; *THES* trichohepatoenteric syndrome; *AGS* Aicardi-Goutières syndrome; *T1D* type I diabetes; *CAD* coronary artery disease; *AD* Alzheimer disease; *MS* multiple sclerosis.

**Table 2:** RBP-mediated regulation of autoimmunity and autoinflammation.

RBPs	mRNA targets	Post-transcriptional regulation (PTR)	Physiological function of RBPs	Genetic mutation or deficiency strategy	Inflammatory or autoimmune phenotype
ADAR1	dsRNA	Adenosine-to-inosine editing	Suppressing IFN signaling	Genetic mutations in human	AGS, with upregulation of IFN expression, upregulation of ISG^63,64^
			Maintaining hematopoietic stem cells (HSCs) and suppressing IFN signaling	Tamoxifen-inducible HSC-specific deficiency in mice (SCL-Cre-ERT)	Embryonic death, global upregulation of type I and II IFN-inducible transcripts and rapid apoptosis in HSCs^63,64^
			Preventing MDA5-dependent immune pathology	P195A point mutation in mice	Lethal inflammatory disease dependent on MDA5, type I IFNs, and the eIF2α kinase PKR^69^
			Preventing MAVS-dependent autoimmune pathology	Point mutation abolishing Z-RNA binding ability in mice	Spontaneous induction of type I IFN in multiple organs dependent on MAVS^70^
SKIV2L	Self dsRNA	3′-to-5′ mRNA degradation	Inhibiting activation of the RIG-I-like receptors	Deficiency in human	Strong type I IFN signature in peripheral blood^72^
			Preventing mTORC1-dependent autoinflammation	Tamoxifen-inducible whole-body deficiency in mice	Skin inflammation and hair abnormality with mTORC1-dependent T cell overactivation^73^
			Preventing mTORC1-dependent autoinflammation	Keratinocyte-specific deficiency in mice (K14-Cre)	Epidermal hyperproliferation with aberrant activation of the mTORC1 pathway^73^
TIA-1	mRNA encoding TNF	Translation silencing	Inhibiting LPS-induced TNF production and inflammation	Deficiency in mice	Hypersensitive to the toxic effects of LPS and chronic arthritis^150,151^
TTP (*ZFP36*)	ARE of mRNAs encoding IL-6, TNF, IL23p19, and IL-17	mRNA degradation	Inhibiting TNF production and IL23-IL17A axis to avoid severe AIDs	Deficiency in mice	Inflammatory phenotype including cachexia, erosive arthritis, dermatitis, conjunctivitis, glomerular mesangial thickening, and high titers of autoantibody, dependent on TNF and IL23-IL17A axis^138,139^
			Inhibiting TNF production to prevent LPS shock	Myeloid deficiency in mice (LysM-Cre)	Increased sensitivity to LPS shock with extensive organ damage and high serum TNF^140^
			Downregulating Th17 function and Th17-mediated inflammation	T cell-specific deficiency in mice (CD4-Cre)	Spontaneous chronic skin inflammation and more severe colitis with increased effector Th17 cells^180^
Regnase-1 (*Zc3h12a*)	Stem loop of mRNAs encoding IL-6, c-Rel, OX40, and IL-2	mRNA decay	Inhibiting IL-6 and IL-12 production to prevent autoimmune disorders	Deficiency in mice	Severe anemia, augmented serum Ig levels and autoantibody production, with a greatly increased inflammatory plasma cell infiltration to the lung, increased production of IL-6 and IL-12p40 in macrophages^142^
			Suppressing T cell activation to prevent autoimmune pathogenesis	T cell-specific deficiency in mice (CD4-Cre)	Early death, spontaneous autoimmune disorders with severe splenomegaly, increased serum Ig and autoantibody levels, enhanced CD4^+^ T cell activation^181^
			Suppressing B cell activation and germinal cell (GC) response	B cell-specific deficiency in mice (Mb1-Cre)	Severe splenomegaly and lymphadenopathy, leukocyte infiltration in the liver, hyperimmunoglobulinemia with aberrant B cell populations^222^
HuR (*Elavl1*)	ARE of mRNAs encoding IL-4, IL-13, TNF, IL-17, Dlst	Inhibiting mRNA translation/stability	Inhibiting inflammatory cytokine mRNA translation to prevent pathologic inflammation and colorectal carcinogenesis	Myeloid deficiency in mice (LysM-Cre)	Pathologic inflammation and colorectal carcinogenesis, associated with an exacerbated proinflammatory cytokine expression and macrophage chemotaxis^149^
		Stabilizing mRNA	Enhancing IL-17 and GM-CSF expression to promote Th17 differentiation and EAE	Activated T cell conditional deficiency in mice (OX40-Cre)	Delayed severity of experimental autoimmune encephalomyelitis (EAE) with decreased Th17 differentiation^193,194^
		Regulating mRNA splicing	Controlling B cell energy metabolism and preventing ROS accumulation upon B cell activation	B cell-specific deficiency in mice (Mb1-Cre)	Defective mitochondrial metabolism and defective B cell proliferation, differentiation and GC response^225^
Arid5a	Stem loop of mRNAs encoding IL-6, OX40, Stat3, and T-bet	Stabilizing mRNA	Enhancing IL-6 production and Th17 response to promote inflammatory response and autoimmunity	Deficiency in mice	Resistance to LPS shock due to reduced IL-6 and IFN-γ levels; Resistance to EAE due to reduced IL-6 levels and Th17 responses^144^
Roquin (*Rc3h1* and *Rc3h2*)	Stem loop of mRNAs encoding ICOS, c-Rel, IRF4, and OX40	mRNA degradation	Repressing Th17 genes to inhibit Th17-depedent inflammation	T cell-specific deficiency in mice (CD4-Cre)	Spontaneous lung inflammatory pathology with enhanced Th17 cell differentiation^183^
			Repressing inappropriate T cell activation, Tfh cell differentiation and systemic inflammation	Sanroque mice with a mutation in the ROQ domain of Roquin (Rc3h1)	Lupus-like autoimmune syndrome with increased autoantibodies, lymphocyte accumulation, excessive Tfh cells and GC response^187,188^
			Repressing inappropriate T cell activation, Tfh cell differentiation and systemic inflammation	T cell-specific Rc3h1 and Rc3h2 deficiency in mice (CD4-Cre)	Lymphadenopathy and splenomegaly with increased spleen weight and cellularity, enhanced T cell activation and Tfh cell differentiation^190,191^
			Repressing inappropriate T cell activation, Tfh cell differentiation and systemic inflammation	Rc3h2 RING domain deficiency in Rc3h1^san/san^ mice	Early lethality and exacerbated tissue inflammatory damage^190^
			Suppressing the PI3K-mTOR signaling to inhibit Th cell differentiation and Treg-to-Tfr conversion	Treg cell-specific Rc3h1 and Rc3h2 deficiency in mice (Foxp3-Cre)	Increased Ig levels, more severe colitis, enhanced Th differentiation and enhanced Treg to Tfr cell conversion^216^
SRSF1	Multiple mechanisms dependent on different PTR events	Alternative splicing, mRNA stability, translation control	Preventing T cell hyperactivity and systemic autoimmunity	T cell-specific deficiency in mice (Lck-Cre)	Systemic autoimmunity and lupus nephritis with T cell hyperactivity dependent on mTORC1 pathway ^250–252^
Mettl3	mRNAs encoding SOCS, TIRAP, CD40 and CD80	m^6^A modification for mRNA degradation	Targeting the IL-7/STAT5/SOCS pathway to control T cell homeostasis	T cell-specific deficiency in mice (CD4-Cre)	Resistance to colitis and blocked T cell proliferation and differentiation^210^
		m^6^A modification for mRNA degradation	Inhibiting SOCS mRNA levels, upregulating the IL-2-STAT5 signaling pathway to maintain Treg functions and stability	Treg cell-specific deficiency in mice (Foxp3-Cre)	Enlarged peripheral lymph nodes and spleen, and severe systemic AID, early death with loss of Treg suppressive function^207^
		Enhancing translation	Promoting DC maturation and activation by enhancing TIRAP, CD40, and CD80 mRNA translation	DC-specific deficiency in mice (CD11c-Cre)	Inhibited maturation, proinflammatory cytokine secretion, T cell stimulating function of DC^169^

## RBPs regulate innate immune response in autoimmunity and autoinflammation

**Fig. 2:**
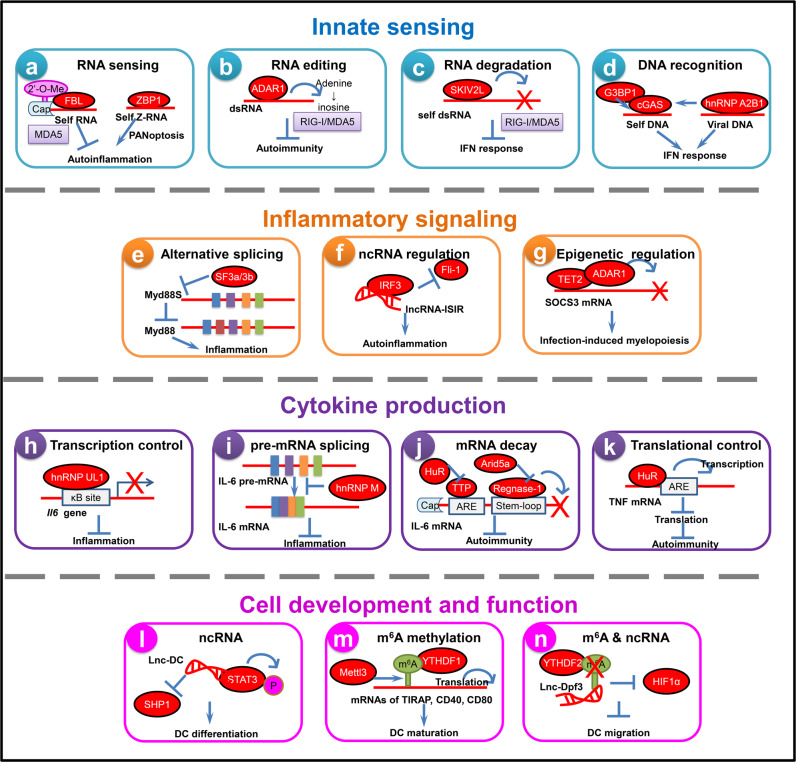
Control of RNA metabolism and function by RBPs in innate immune response during autoimmunity and autoinflammation. RBPs regulate the activation of PRR-triggered innate immunity via targeting various steps at transcriptional, post-transcriptional or translational levels. **a** FBL mediates 2′-*O*-methylation of RNA and prevents the innate recognition by MDA5 and IFN responses. ZBP1 recognizes self Z-RNA to promote pathologic inflammation. **b** ADAR1 mediates RNA A-to-I editing, thus allowing effective antiviral immunity while preventing pathogenic autoinflammation. **c** The SKIV2L subunit of the RNA exosome cleaves self RNA produced by the endonuclease IRE-1, and thereby inhibits RIG-I activation and type I IFN-dependent autoinflammation. **d** G3BP1 enhances DNA binding of cGAS and cGAS-dependent IFN production. hnRNP A2B1 recognizes viral DNA and enhances cGAS/STING-dependent IFN response. **e** The short isoform of MyD88 (MyD88s) inhibits TLR-triggered inflammation due to its failure to recruit IRAK-4. SF3a and SF3b mRNA splicing complexes reduce the MyD88s mRNA levels. **f** lncRNA-ISIR binds to IRF3 and impedes the inhibitory effect of Fli-1, thus enhancing IRF3 activation, IFN response and autoinflammation. **g** TET2 promotes the degradation of SOCS3 mRNA through ADAR1, facilitating cytokine-induced emergency myelopoiesis and mast cell expansion and activation during pathogen infection. **h** hnRNP UL1 inhibits NF-κB-mediated inflammation via competing with NF-κB to bind κB sites, while hnRNP UL1 expression decreases in RA patients. **i** hnRNP M inhibits pre-mRNA splicing and maturation of inflammatory transcripts such as IL-6 to negatively regulate inflammatory responses. **j** TTP and Regnase-1 destabilize mRNAs of proinflammatory cytokines such as IL-6 and TNF to control autoimmunity. **k** HuR downregulates mRNA translation to suppress aberrant inflammation and autoimmunity. **l** lnc-DC controls human DC differentiation and function via directly binding to STAT3 in the cytoplasm to prevent SHP1 binding and promote STAT3 phosphorylation. **m** Mettl3 mediates m^6^A methylation of transcripts of co-stimulatory molecules CD40, CD80 and TLR4 signaling adaptor TIRAP to enhance their translation in DCs, stimulating T cell activation and strengthening TLR4/NF-κB signaling-induced cytokine production. **n** CCR7 ligation upregulates expression of lnc-Dpf3 via relieving m^6^A-dependent degradation, consequently leading to inhibition of HIF1α-dependent glycolysis and DC migration. RBPs responsible for each step are shown in red ovals. FBL fibrillarin, ZBP1 Z-DNA-binding protein 1, MDA5 melanoma differentiation-associated gene 5, ADAR1 adenosine deaminase acting on RNA 1, RIG-I retinoic acid-inducible gene I, SKIV2L superkiller viralicidic activity 2-like, G3BP1 GTPase-activating protein SH3 domain-binding protein 1, cGAS cyclic GMP AMP synthase, hnRNA A2B1 heterogeneous nuclear ribonucleoprotein A2B1, MyD88 Myeloid differentiation primary response gene 88, SF3a/3b splicing factor 3a/3b, IRF3 interferon regulatory factor 3, Fli-1 flightless-1, TET2 ten-eleven translocation 2, hnRNP UL1 heterogeneous nuclear ribonucleoprotein UL1, hnRNP M heterogeneous nuclear ribonucleoprotein M, TTP tristetraprolin, HuR human antigen R, Arid5a AT-rich interactive domain-containing protein 5a, ARE adenine uridine (AU)-rich elements, DC dendritic cells, STAT3 signal transducer and activator of transcription 3, SHP1 Src-homology 2 (SH2) domain-containing phosphatase 1, Mettl3 methyltransferase like 3, YTHDF1/2 YTH domain-containing protein 1/2, HIF1α hypoxia-inducible factor-1 alpha.

Accumulating evidence has revealed disturbed innate immune cell accumulation and function in autoimmune responses. Activation of pattern recognition receptors (PRRs) results in profound activation of innate signaling, leading to immune cell activation and inflammatory responses. These events further recruit and activate inflammatory cells, causing breakdown of immune tolerance and generation of autoinflammation and tissue injury.^32,33^ In this section, we will discuss how RBPs and RNA metabolism affect various stages of the innate immune response, including (1) innate sensing, (2) inflammatory signaling, (3) proinflammatory cytokine production and (4) innate immune cell development and function, to regulate autoimmune responses and inflammatory pathologies (Fig. 2).

### RBPs regulate innate immune sensing in autoimmunity and autoinflammation

The recognition of pathogenic antigens by PRRs, such as Toll-like receptors (TLRs), retinoic acid-inducible gene I (RIG-I)-like receptors (RLRs), nucleotide-binding oligomerization domain (NOD)-like receptors (NLRs), generally activates inflammatory response and protective immune defense. In contrast, the recognition of self-antigens by innate sensors is considered as initial triggering events in autoimmune inflammatory responses. In particular, the recognition of self nucleic acids derived from apoptotic cells or neutrophil extracellular traps (NETs) by endosomal and cytosolic innate sensors, such as endosomal TLRs (TLR3/7/8/9) and cytosolic RLRs (RIG-I and melanoma differentiation-associated protein 5 (MDA5)), of plasmacytoid dendritic cells (pDCs), dendritic cells (DCs), and antigen-specific B cells is essential for lupus autoimmunity.^34–36^ We recently reveal a RNA sensing-independent role of nuclear RIG-I in the induction of cellular apoptosis. Nuclear RIG-I is present in peripheral blood mononuclear cells (PBMCs) from SLE patients, implying a non-canonical role for nuclear RIG-I in mediating the autoimmune pathology of SLE.^37^ It is therefore critical to understand the molecular mechanism that enables specific detection of nucleic acid of different origins.^38^

#### RNA sensing

RNA sensing is a critical mechanism utilized by RBPs to enable innate sensors to distinguish between pathogenic and self RNAs. 2′-*O*-methylation at the N1 position in capped mRNA serves as a specific signature of self RNAs to prevent their recognition by MDA5.^39^ Interferon (IFN)-induced protein with tetratricopeptide repeats 1 (IFIT1) is an antiviral RBP that recognizes viral RNAs lacking 2′-*O*-methylation, thus preventing active translation and inhibiting viral replication.^40,41^ We recently show that RNA 2′-*O*-methyltransferase fibrillarin (FBL)-mediated 2′-*O*-methylation of RNA prevents the innate recognition of these RNAs by MDA5, thereby inhibiting IFN responses and facilitating virus entry into macrophages. Once FBL is downregulated, the decreased 2′-*O*-methylation modifications of RNA in macrophages are recognized as “non-self” RNA by MDA5, leading to autoinflammation by inducing the expression of type I IFN and IFN-stimulated genes (ISGs).^42^ Therefore, 2′-*O*-methylation is a critical mechanism governing innate sensing between self and non-self RNA. The immunological relevance of 2′-*O*-methylation on other RNA targets such as ribosomal RNAs (rRNAs), transfer RNAs and small nucleolar RNAs remains to be further revealed. *N*^6^-methyladenosine (m^6^A) RNA modification is also identified as a mark for innate immune discrimination of self from non-self RNA. The m^6^A-modified circular RNA (circRNA) evades host detection as non-self RNA through binding to the reader YTH domain-containing family protein 2 (YTHDF2) and loses the ability of unmodified circRNA in activating RIG-I/MAVS/IRF3/IFN pathway.^43^ circRNAs have been shown to negatively regulate dsRNA-dependent protein kinase R (PKR) activation and autoimmunity; consistently, circRNA reduction and augmented PKR phosphorylation are found in SLE patients.^44^ Given the role of 2′-*O*-methylation and m^6^A in marking RNA as self, targeting these modifications may serve as potential strategy to enhance mRNA vaccine efficacy.^45,46^

Inflammasomes are large protein platforms triggered by various internal or external stimuli via NLR members, to promote caspase-1-dependent cleavage and maturation of inflammatory cytokines IL-1β and IL-18, as well as to induce gasdermin D-induced pyroptotic cell death.^47^ Overactivation of inflammasome is closely related to a variety of inflammatory and autoimmune diseases, such as diabetes,^48^ Alzheimer’s disease^49^ and atherosclerosis.^50,51^ RBPs are critical regulators of inflammasome activation and inflammatory disease development. Z-DNA-binding protein 1 (ZBP1) is a RBP capable of sensing both DNA and RNA viruses and mediating antiviral effects via inducing inflammatory cell death involving pyroptosis, apoptosis, and necroptosis (PANoptosis).^52,53^ Importantly, ZBP1 can be activated by endogenous nuclear acids in the absence of viral infection. Ablation of the Zα2 domain of ZBP1 can rescue perinatal lethality and skin inflammation caused by receptor-interacting protein kinase 1 (RIPK1) mutation, indicating that sensing of endogenous Z-RNA by ZBP1 is involved in pathological inflammation and the development of chronic inflammatory pathologies.^54^ ZBP1 and necroptosis are upregulated in patients with inflammatory bowel disease (IBD) and can promote the development of bowel inflammation, suggesting that ZBP1-dependent necroptosis is involved in the pathogenesis of IBD and that targeting necroptosis may serve as a potential approach for the treatment of IBD.^55^ PKR is also essential for inflammasome activation via physically interacting with several inflammasome components such as NLRP3, NLRC4, and AIM2.^56^ On the contrary, ADAR1 could suppress PANoptosis by interacting with the Zα2 domain of ZBP1, which subsequently promotes tumorigenesis.^57^ Detailed contributions of the inflammasome-interacting RBP networks involving ZBP1, ADAR1, PKR, as well as some lncRNAs^58,59^ in the development of AIDs will provide new insights into inflammasome activation and suggest potential targets to treat inflammation.

#### RNA editing

ADAR1, which mediates adenosine-to-inosine (A-to-I) editing of RNA, has been implicated in various infectious and autoimmune diseases, such as RA and systemic sclerosis.^60–62^ Mutations in *ADAR1* cause AGS, an autoinflammatory disorder associated with high levels of autoantibodies and type I IFNs.^63,64^ ADAR1-mediated A-to-I editing marks endogenous dsRNAs as self and prevents their recognition by MDA5.^65,66^ ADAR1 edits Alu elements in Pol II-transcribed mRNAs and thus prevents endogenous RNAs from activating PKR to avoid autoinflammation.^67^ Unlike wild-type MDA5 that is inefficient in recognizing Alu:Alu hybrids under A-to-I editing, a gain-of-function variant of MDA5 in AGS can recognize Alu:Alu hybrids to trigger antiviral immune response.^68^ Therefore, ADAR1-dependent RNA editing allows effective antiviral immunity while preventing pathogenic autoinflammation. Further studies are focusing on the detailed molecular basis for ADAR1-dependent regulation of autoimmunity. Mice with the P195A mutation of ADAR1 develop lethal AGS-like diseases with the eIF2α kinase PKR and the integrated stress response downstream of eIF2α phosphorylation as key drivers of immunopathology.^69^ In another strain of mice with an ADAR1 mutant without the Z-form RNA binding ability, mitochondrial antiviral signaling protein (MAVS)-dependent IFNs and ISGs are spontaneously induced in multiple organs, which is associated with significant changes in A-to-I editing of transposable elements.^70^ These findings shed light on how ADAR1 prevents autoimmunity and propose new therapeutic target for the treatment of human diseases associated with the ADAR1 dysfunction.

#### RNA degradation

The super-killer (SKI) complex component *SKIV2L*, encoding a subunit of the RNA exosome responsible for RNA degradation, is a potential susceptibility gene for human SLE.^71^ Mutations in the human *SKIV2L* gene cause tricohepatoenteric syndrome characterized by immunodeficiency, severe diarrhea, skin and hair abnormalities. Notably, the peripheral blood of SKIV2L-deficient human has a strong type I IFN signature. *SKIV2L*-knockdown macrophages exhibit elevated RIG-I activation and type I IFN production induced by endogenous self RNAs generated by the endonuclease inositol-requiring enzyme 1 (IRE1) during unfolded protein response (UPR).^72^ Therefore, SKIV2L is critical for suppressing RIG-I signaling via mediating degradation of self RNA. However, SKIV2L-deficient mice display skin-specific autoinflammation associated with hyperproliferation of keratinocytes and overactivation of T cells, but independently of IFN. The mechanistic target of rapamycin complex I (mTORC1) signaling is responsible for epidermal hyperplasia and skin inflammation in SKIV2L-deficient mice. Treatment of SKIV2L-deficient mice with the mTOR inhibitor rapamycin relieves their skin inflammation, suggesting a possible therapeutic strategy for SKIV2L-associated trichohepatoenteric syndrome.^73,74^ These studies indicate a context-dependent immune regulation function of SKIV2L. SKIV2L has recently been shown to be universally recruited by ribosomes to mediate mRNA translation surveillance.^75^ It is likely that SKIV2L prevents mRNA from aberrant ribosome translation to avoid overactivation of mTORC1 under homeostatic conditions, while degrades or cleaves the immunogenic self or viral RNA to avoid excessive IFN responses under stress conditions.

The 2′-5′ linked oligoadenylates (2-5A), which are generated by oligoadenylate synthetase (OAS) upon dsRNA sensing, can bind ribonuclease L (RNase L) and activate its dimer formation, resulting in degradation of viral and cellular ssRNAs and restriction of viral infection.^76^ RNase L cleaves self-RNA to produce small RNA products which trigger IFN production and antiviral immunity via RIG-I, MDA5 and IPS-1 pathways.^77^ RNase L also catalyzes the generation of RNA cleavage products to trigger NLRP3 inflammasome activation and enhance IL-1β production during viral infections.^78^ Apart from their potent antiviral effects, RNase L is increasingly implicated in autoimmunity-related processes such as apoptosis, autophagy, cell migration, inflammation.^79^ RNase L activation promotes cell death in ADAR1-deficient human lung cell line,^80^ and a phenolic small-molecule inhibitor of RNase L has been recently reported to prevent cell death from ADAR1 deficiency.^81^ Further studies are required to determine the roles of RNase L in other forms of cell death and their contribution to the development of autoimmunity and autoinflammation.

#### DNA recognition

Cytosolic DNA, derived from either invading viruses or endogenous chromosomal or mitochondrial products, can be recognized by the cytosolic DNA sensor cyclic GMP, AMP synthase (cGAS), leading to the stimulator of interferon genes (STING)/TANK binding kinase 1 (TBK1)/IFN regulatory factor (IRF)-dependent IFN production.^82^ Three prime repair exonuclease 1 (TREX1) is a cytoplasmic exonuclease that mediates DNA degradation, and mutations of *TREX1* are associated with human inflammatory diseases such as AGS and SLE.^83,84^ Abnormal DNA accumulation due to inhibition of TREX1 or DNaseII has been found to activate the cGAS/STING/IRF3 pathway, which enhances the autoantibody production and autoimmune pathogenesis.^85,86^ cGAMP and cGAS elevations are observed in a subset of patients with SLE and correlate with the disease activity.^87^ Moreover, tumor necrosis factor (TNF) stimulation induces mtDNA release into the cytosol, which is recognized by cGAS to trigger IFN responses and inflammatory arthritis. Deficiency of cGAS in mice blocks IFN responses and relieves the autoimmune pathologies such as inflammatory cell infiltration and joint swelling in inflammatory arthritis.^88^ Therefore, cGAS-dependent DNA recognition is essential for IFN response and many forms of autoimmunity and autoinflammation.

Interestingly, increasing evidence suggests that the cGAS/STING pathway can also respond to RNA virus infections.^89–91^ Complex interactions between cGAS and known RBPs have been implicated in inflammation and autoimmunity. G3BP1 (GTPase-activating protein SH3 domain-binding protein 1), a RBP important for stress granule assembly,^92^ enhances DNA binding of cGAS and cGAS-dependent IFN production, which is associated with enhanced intracellular nucleic acid-induced autoimmunity.^93^ The G3BP1 chemical inhibitor EGCG can specifically inhibit cGAS-related autoinflammation.^94^ These studies indicate a potential role for RNA- or RBP-dependent activation of cGAS pathway in autoimmunity. We show that the RBP heterogeneous nuclear ribonucleoprotein A2B1 (hnRNP A2B1) can recognize viral DNA to initiate type I IFN production in a TBK1-STING-dependent way as well as enhance cGAS expression and cGAS-initiated IFN production, thus contributing to the antiviral defense.^95^ hnRNP A2B1 can also recognize host genomic DNA, and autoantibodies against hnRNP-A2 are observed in patients with SLE,^96^ indicating a potential involvement of hnRNP A2B1 in the development of autoinflammation. The detailed mechanisms for RBP-related recognition of pathogenic versus self DNA and its relevance in the development of antiviral immunity or autoimmune responses require further investigations.

### RBPs regulate innate inflammatory signaling in autoimmunity and autoinflammation

Ligation of PRRs triggers intracellular signaling networks that converge on the activation of key transcriptional factors nuclear factor kappa B (NF-κB) and IRFs for the induction of transcriptional activation of proinflammatory cytokines and IFNs, respectively.^97^ RBP-mediated post-transcriptional, translational and post-translational regulation of a variety of signaling adaptors and regulators is important in modulating the innate signaling and autoimmunity.

#### Alternative splicing

Alternative splicing is triggered by various stress signals and represents an important RBP-dependent post-transcriptional regulatory mechanism that affects many molecular components of inflammation and autoimmunity, including receptors, adaptors, and proinflammatory cytokines.^98^ Myeloid differentiation primary response gene 88 (*Myd88*) encodes an important adaptor protein (MyD88) associated with TLR signaling and is widely implicated in immune pathogenesis and tissue damage of AIDs. Signaling through MyD88 has been shown to be essential for the development of autoimmune nephritis in MRL/lpr mice.^99^ The polymorphism rs6853 of *MYD88* is associated with inflammatory response and RA development in a Brazilian cohort.^100^ MyD88 plays dual roles in regulating intestinal inflammation. MyD88 on one hand maintains intestinal epithelial integrity and homeostasis,^101,102^ but on the other hand promotes TNF-independent intestinal inflammation and epithelial tissue damage caused by A20 and ABIN-1 deletion.^103^ A short isoform of MyD88 (MyD88s), encoded by an alternatively spliced mRNA, has been shown to inhibit the full-length form of MyD88 and thus dampen the downstream activation of TLR signaling and inflammatory response.^104,105^ How the altered splicing of MyD88 is specifically involved in the development of autoimmunity requires further investigations.

#### ncRNA regulation

RBPs can widely interact with ncRNAs to regulate cellular processes such as gene transcription, RNA stability and protein function.^106–108^ Multiple miRNAs have been shown to negatively regulate innate immune signaling via targeting key signaling molecules in NF-κB and IRF pathways.^109^ These regulations are important for the maintenance of immune system homeostasis and the prevention of excessive inflammatory response. Dysregulations of miRNAs have been closely associated with autoimmunity and some miRNAs are potential biomarkers or therapeutic targets for AIDs.^110,111^ For example, reduced expression of miR-23b results in higher expression of TGF-beta-activated kinase 1 binding protein 2 (TAB2), TGF-beta-activated kinase 1 binding protein 3 (TAB3) and I kappaB kinase α (IKKα), and increased production of TNFα, IL-1β and IL-17 in patients with AIDs such as SLE and RA.^112^ miR-23a/b also suppresses cGAS-mediated autoimmunity via interacting with the 3′ UTR of the cGAS mRNA.^113^ In addition, miR-146a can target IRF5 and STAT-1 to inhibit the type I IFN pathway in human lupus while type I IFNs can inhibit miR-146a maturation and thus contribute to SLE pathogenesis.^114,115^ miR-146a also inhibits the proliferation of synovial fibroblasts and prevents joint damage in arthritis via targeting TNF receptor associated factor 6 (TRAF6).^116^ Therefore, miRNAs are important for regulating local and systemic autoimmunity in a cell type-specific manner. Whether interactions between miRNAs and their mRNA targets are regulated by RBPs in the context of autoimmunity remains to be determined.

We identified various lncRNAs as critical regulators of IFN-dependent autoinflammation and antiviral immunity via interacting with either canonical or non-canonical RBPs. For example, IRF3-binding lncRNA-ISIR is positively correlated with type I IFN levels and disease severity of human lupus, and strengthens IRF3 activation and IFN production in viral infection and autoinflammation;^117^ the lncRNA Malat1 is reduced in PBMC of SLE patients and plays a role in inhibiting autoinflammatory interferonopathies by targeting the transactive response DNA-binding protein 43 (TDP43);^118^ the IFN-independent lncRNA-ACOD1 promotes viral replication by directly binding the metabolic enzyme glutamic-oxaloacetic transaminase 2 (GOT2) to enhance its catalytic activity.^119^ These studies highlight the important role of ncRNA–RBP interactive network in the regulation of innate responses and autoimmune pathogenesis.

#### Epigenetic regulation

Many epigenetic enzymes or modulators have been shown to regulate mRNAs encoding signaling activators or suppressors for a coordinated modulation of gene expression and signal transduction. For example, the epigenetic enzyme ten-eleven translocation 2 (TET2) binds to the 3′ UTR of the suppressor of cytokine signaling protein 3 (SOCS3) mRNA and promotes its degradation through ADAR1. SOCS3 is a suppressor of the JAK-STAT signaling pathway; TET2-mediated degradation of SOCS3 mRNA promotes cytokine-induced emergency myelopoiesis and mast cell expansion during pathogen infection.^120^ TET2 also recruits histone deacetylases HDAC1/2 to repress transcription of *Il6* and *Il1β* via histone deacetylation in innate myeloid cells including DCs and macrophages, thus mediating inflammation resolution.^121^ The identification of RNA-binding capacity and potential RNA-binding site of TET2 suggests TET2 as a potential RBP important for inflammation resolution and infection-induced myelopoiesis.^122^ The differential recognition of RNA and DNA by epigenetic modulators and their interactions with known RBPs add another layer of epigenetic regulation of innate immunity and inflammation worthy of further investigations.

### RBPs regulate the production of proinflammatory cytokines in autoimmunity and autoinflammation

Activation of innate inflammatory signaling leads to the production of large amounts of proinflammatory cytokines such as IL-6, IL-1β, IL-18, TNF, and type I IFNs that are essential mediators of autoimmune pathogenesis. Recent scRNA-seq analysis reveals the elevated expression of ISGs in monocytes in SLE patients^123,124^ and IL-6 expression in THY1^+^HLA-DRA^hi^ fibroblasts and IL-1β expression in pro-inflammatory monocytes in RA patients.^125–127^ It is critical to understand how RBP–RNA interactions determine the specific transcriptional signatures of innate inflammatory cytokines in distinct immune cells in different autoimmune processes.

#### Transcriptional control

Transcriptional control of innate inflammatory cytokines is precisely regulated by coordinated networks of transcriptional regulators, epigenetic enzymes and ncRNAs.^128,129^ Importantly, RBPs can interact with both DNA and RNA, contributing to functional integration of transcriptional and post-transcriptional machineries. Some nuclear RBPs are widely present in active chromatin regions and are associated with transcription factors at gene promoters, thus enhancing gene transcription.^130^ We recently show that hnRNP UL1 inhibits NF-κB-mediated inflammation via competing with NF-κB on κB binding sites, indicating a splicing-independent role of hnRNP UL1 in restraining inflammatory cytokine expression at the transcriptional level. The expression of hnRNP UL1 is reduced in RA patients, suggesting a strong correlation between decreased hnRNP UL1 level and inflammatory autoimmune disease and proposing a potential therapeutic strategy for controlling aberrant autoinflammation.^131^ It will be important to further clarify how the structural and functional networks among transcription factors, RBPs, RNAs, and DNAs are coordinated in a cell type-specific manner during specific autoimmune or autoinflammatory responses.

#### pre-mRNA splicing

pre-mRNA splicing is an important mechanism for RBP-dependent regulation of proinflammatory cytokine production. Alternative splicing of mRNAs of IL-6 and its receptor IL-6R has been implicated in the pathogenesis of RA.^132–134^ The splicing factor hnRNP M is shown to be a negative regulator of inflammatory and antimicrobial genes in innate immune cells by preventing the maturation of transcripts encoding proinflammatory molecules such as IL-6 via blocking pre-mRNA splicing. Innate stimuli such as LPS induce the phosphorylation of hnRNP M via p38 signaling and release it from inhibiting IL-6 pre-mRNA splicing and expression. hnRNP M associates with the *Il6* genomic locus in the nucleus in an RNA-dependent manner, suggesting an essential role of chromatin–RBP–RNA complexes in shaping transcription complexity during innate immune response.^135^ It will be important to further elucidate the mechanisms underlying the location, interaction and function of chromatin–RBP networks in innate immunity and autoinflammation.

#### mRNA degradation or stabilization

It has been widely shown that RBPs control the expression of proinflammatory cytokines by regulating the stability of mRNAs. For example, TTP (also known as ZFP36) destabilizes mRNAs of proinflammatory cytokines such as IL-6 and TNF via directly binding to ARE and recruiting deadenylation and decapping complexes for mRNA decay.^136,137^ Mice deficient of TTP display severe autoimmune phenotypes such as cachexia, erosive arthritis, and dermatitis, which can be abolished by anti-TNFα antibody or combined deficiency of IL-23.^138,139^ Mice with myeloid-specific TTP deficiency have minimal autoimmune inflammation but are highly sensitive to LPS shock with high serum TNF.^140^ TTP is also expressed in atherosclerotic lesions both in human and mice, and functionally inhibits the expression of pro-inflammatory mRNA transcripts.^141^ Thus, controlling the stability of mRNAs encoding innate proinflammatory cytokines such as TNF and IL-23 by TTP is critical for preventing severe inflammation.

Another well-recognized RBP that controls the mRNA stability during autoimmune responses is Regnase-1. Mice deficient of Regnase-1 exhibit severe autoimmune disorders characterized by augmented serum immunoglobulin and autoantibody levels, splenomegaly and pulmonary inflammation.^142^ Mechanistically, Regnase-1 recognizes the stem loop in mRNAs encoding proinflammatory cytokines, such as IL-6, to mediate mRNA decay with its RNase activity. IκB kinase complex mediates ubiquitination and degradation of Regnase-1, thereby relieving its inhibition of IL-6 mRNA expression.^143^ Consistently, mice deficient of Arid5a, a RBP that inhibits Regnase-1 function and thus stabilizes the IL-6 mRNA, have reduced IL-6 and TNF serum levels and are resistant to the development of autoimmune pathogenesis in experimental autoimmune encephalomyelitis (EAE).^144^ Interestingly, cytoplasmic polyadenylation element binding protein 4 (CPEB4) is shown to stabilize anti-inflammatory mRNAs such as SOCS and consequently promote inflammation resolution program.^145^ How these opposite actions of mRNA decay or stabilization processes coordinately determine the transcription dynamics of pro- and anti-inflammatory genes and orchestrate inflammation initiation and resolution programs in autoimmunity remains to be fully addressed.

#### Translational control

Similar to Arid5a-mediated transcript stabilization, HuR is proposed to act as a stabilizer of inflammatory mRNAs bearing ARE via antagonizing their binding by destabilizing RBPs such as TTP.^146,147^ On the other hand, however, HuR also negatively affects mRNA translation through synergizing with the translational silencer T-cell restricted intracellular antigen-1 (TIA-1) during inflammation.^148^ Mice with myeloid-specific deficiency of HuR exhibit pathologic inflammation and colorectal carcinogenesis, which is associated with an exacerbated proinflammatory cytokine expression due to a lack of inhibitory effects on their translation and/or stability.^149^ Therefore, the translational silencing by HuR may be dominant over mRNA stabilization in myeloid cells for the suppression of aberrant inflammation and autoimmunity. In addition, TIA-1 mediates translational silencing of mRNA encoding TNF, thus reducing the sensitivity of mice to acute LPS shock or inflammatory arthritis.^150,151^ Downregulation of *TIA-1*/*TIAR* genes is observed in ulcerative colitis patients by transcriptome meta-analysis and might contribute to the enhanced IL-1β production during autoimmunity.^152^

These RBP-dependent regulations of splicing, stability and translation of mRNAs encoding cytokines are essential for the prevention or promotion of aberrant chronic inflammation or harmful autoimmunity. Meanwhile, how these post-transcriptional or translational regulations affect biological functions or immune regulatory properties of cytokines remains to be investigated.

### RBPs regulate the innate immune cell development and function in autoimmunity and autoinflammation

In addition to production of proinflammatory cytokines, innate immune cells carry out many other cellular functions, which are important for immune homeostasis and tissue integrity. Innate immune sensing and activation of innate immune signaling can also influence the development and function of innate immune cells, such as DC maturation, macrophage polarization, natural killer (NK) cell activation, resulting in diversified immunological effects and regulation of autoimmunity.

#### lncRNA

lncRNAs display lineage-specific expression pattern and play essential roles in determining functional hematopoietic differentiation.^153^ For example, HOTAIRM1 is critical for myeloid cell differentiation in human leukemia cells via regulating the expression of genes associated with granulocyte activation, maturation, and defense response.^154^ Later studies suggest potential role of HOTAIRM1 in regulating differentiation and function of myeloid-derived suppressor cell (MDSC) during viral infection.^155,156^ We show that the lncRNA lnc-DC controls human DC differentiation and function via directly binding to signal transducer and activator of transcription 3 (STAT3) in the cytoplasm to promote STAT3 phosphorylation.^157^ Further studies identify lnc-DC as a potential biomarker for AIDs such as MS and primary Sjögren’s Syndrome.^158,159^ Macrophages effectively eliminate dying cells via efferocytosis, and failure in efferocytosis is one of important drivers of the progression of atherosclerosis.^160,161^ HuR is shuttled into cytosol in the absence of macrophage-specific lncRNA MAARS (macrophage-associated atherosclerosis lncRNA sequence), where HuR performs RNA-stabilizing functions on a set of apoptosis genes such as *p53* and *p27*, to decrease macrophage apoptosis and increase efferocytosis, consequently preventing atherosclerosis progression.^162^ The detailed relationships between these lineage-specific lncRNAs and autoimmunity are worthy of further investigations.

#### m^6^A methylation

m^6^A modification of mRNAs plays broad roles in immune cell development and function via regulating mRNA biogenesis, metabolism and function. Although the role of m^6^A modification of mRNAs in tumor and viral infection has been intensively studied, its association with autoimmunity is less understood.^163^ Genetic mutations in the m^6^A writer *METTL3* (methyltransferase like 3) correlate with increased susceptibility to autoimmune thyroid disease.^164^ mRNA levels of a set of m^6^A-related proteins, such as the writer methyltransferase-like 14 (METTL14), the eraser AlkB homolog 5 (ALKBH5), and the reader YTHDF2, are decreased in SLE patients, and the levels of ALKBH5 and YTHDF2 mRNAs appear to be associated with disease pathogenesis such as the accumulation of antibodies against dsDNAs and nucleosomes.^165,166^ m^6^A modifications are also critical regulators of vascular inflammation via distinct effects in macrophages, smooth muscle cells or endothelial cells.^167^

m^6^A RNA methylation has been closely related with the cell development of hemopoietic system, nervous system, reproductive system, and immune system.^168^ Development and functional maturation of DCs are important in linking innate immunity with adaptive immunity. Our study shows that Mettl3 can enhance the mRNA translation of TLR4 signaling adaptor TIRAP and co-stimulatory molecules CD40 and CD80 in DCs, contributing to DC maturation and activation.^169^ CCR7 ligation upregulates expression of lnc-Dpf3 via relieving m^6^A-dependent degradation, leading to inhibition of HIF1α-dependent glycolysis and DC migration.^170^ Considering the critical roles of DC function and migration in both immune activation and homeostasis, these mechanisms may offer potential links between m^6^A-dependent post-transcriptional or translational regulations and the control of autoimmunity.

The functional maturation or polarization of macrophages is extensively involved in innate immunity and autoimmune processes. The m^6^A writer METTL3 contributes to the increased severity and development of age/diet-related non-alcoholic fatty liver disease and obesity via promoting macrophage metabolic reprogramming and inflammatory function by controlling damage inducible transcript 4 (DDIT4) mRNA.^171^ Interestingly, METTL3 also reduces susceptibility to bacterial infection and tumor growth in mice, via regulating macrophage activation and reprogramming.^172,173^ We show that m^6^A reader YTHDF3 could suppress the antiviral activity of macrophage by binding to the translation initiation region of FOXO3 mRNA to promote its translation.^174^ It will be interesting to further elucidate how m^6^A could selectively regulate distinct immune cell differentiation or maturation in different immune pathological conditions such as autoimmunity, infection or cancer.

Moreover, NK cells exhibit protective roles in limiting inflammation via killing autoreactive immune cells, but can also promote the initiation and progression of autoimmunity via cytokine production or apoptosis induction.^175^ The cytotoxic activity of NK cells is regulated by m^6^A in the context of antiviral and antitumor immunity. The m^6^A writer METTL3 is required for antitumor effects of NK cells and positively regulates accumulation, effector function and homeostasis of NK cells in a manner involving m^6^A modification of SHP2 coding gene.^176^ Similarly, the m^6^A reader YTHDF2 maintains homeostasis, maturation, antitumor and antiviral immunity of NK cells, by forming a STAT5-YTHDF2 positive feedback loop.^177^ More evidences are required to uncover whether m^6^A methylation in NK cells is associated with autoimmunity and autoinflammation.

## RBPs regulate adaptive immune response in autoimmunity and autoinflammation

**Fig. 3:**
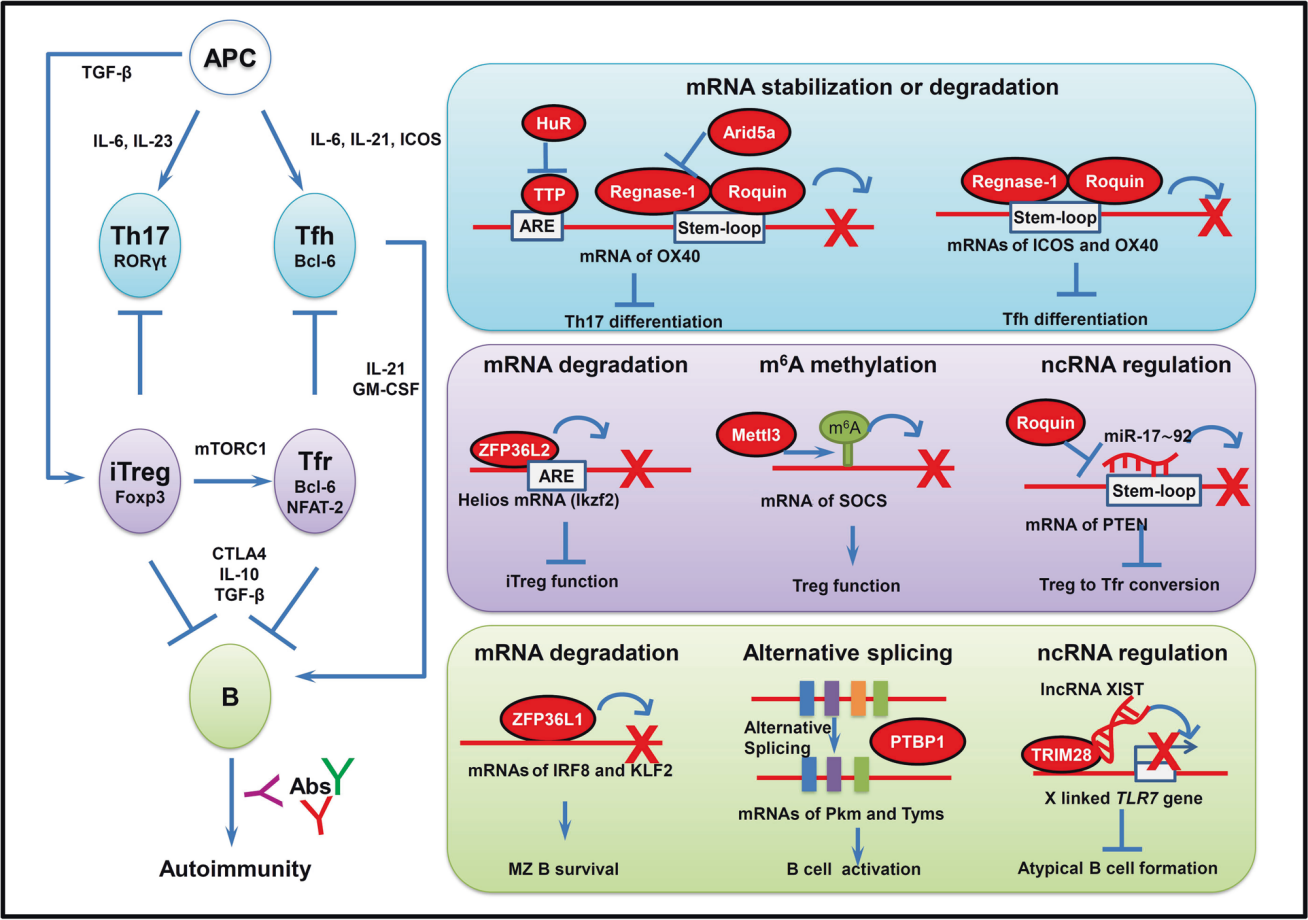
Control of RNA metabolism and function by RBPs in adaptive immune response during autoimmunity and autoinflammation. RBPs regulate the Th cell differentiation, Treg cell function and B cell activation during development of AIDs via multiple mechanisms. Upper: RBP regulation of Th17 and Tfh cell differentiation. TTP, Regnase-1 and Roquin negatively regulate a set of Th17-related genes such as *OX40* via mRNA decay to inhibit Th17 cell differentiation. Regnase-1 and Roquin repress the expression of ICOS and OX40 mRNAs via post-transcriptional regulation to inhibit Tfh cell differentiation. Middle: RBP regulation of Treg cell generation and function. ZFP36L2 decreases Helios expression in Foxp3^+^ Tregs via directly binding to the 3′ UTR of Helios mRNA and destabilizing it, leading to inhibition of iTreg function. Mettl3 mediates m^6^A modification of SOCS mRNA to maintain Treg cell generation and immune tolerance. Roquin upregulates PTEN mRNA expression through antagonizing miR-17–92 binding to PTEN mRNA, and thus suppresses the conversion of Treg to Tfr cells. Bottom: RBP regulation of B cell activation and germinal cell (GC) responses. ZPF36L1 post-transcriptionally limits mRNA levels of transcription factors KLF2 and IRF8 to promote GC response. PTBP1 promotes B cell proliferation and activation in GC via controlling alternative splicing of transcripts of c-Myc target genes such as *Pkm* and *Tyms*. lncRNA XIST interacts with TRIM28 to maintain X-inactivation and inhibit X-linked *TLR7* gene expression, contributing to restraining of atypical B cell formation. RBPs responsible for each of these steps are shown in red ovals. APC antigen-presenting cells, Th17 IL-17-expressing T cells, Tfh follicular T helper cells, Treg regulatory T cells, iTreg inducible regulatory T cells, Tfr follicular regulatory T cells, TTP tristetraprolin, HuR human antigen R, Arid5a AT-rich interactive domain-containing protein 5a, STAT3 signal transducer and activator of transcription 3, IL-17 interleukin 17, ICOS inducible T cell costimulator, ARE adenine uridine (AU)-rich elements, ZFP36L2 zinc finger protein 36, C3H type-like 2, Ikzf2 IKAROS Family Zinc Finger 2, Mettl3 methyltransferase like 3, SOCS suppressor of cytokine signaling protein, PTEN phosphatase and tensin homolog, ZPF36L1 zinc finger protein 36, C3H type-like 1, IRF8 interferon regulatory factor 8, KLF2 kruppel like factor 2, MZ marginal zone, PTBP1 polypyrimidine tract binding protein 1, Pkm Pyruvate Kinase M, Tyms thymidylate synthetase, TRIM28 tripartite motif containing 28.

Dysregulations of T cell- and B cell-dependent adaptive immunity play indispensable roles in the development of autoimmunity and tissue injuries. The long-lasting stimulation by autoantigens and aberrant innate inflammatory responses not only lead to aberrant T cell activation and infiltration in the inflamed tissue, but also strengthen B cell activation and increase the production of autoantibodies. The long-lived auto-reactive memory T and B cells mediate efficient responses to autoantigens and contribute to sustained autoimmunity and chronic inflammation^3,4,5,178^ In this section, we will discuss how RBP–RNA interactions affect the differentiation, function, activation or memory response of adaptive immune cells, whose dysregulations are critical for autoimmune pathogenesis (Fig. 3).

### RBPs regulate the differentiation and function of helper T (Th) cells in autoimmunity and autoinflammation

Th cells such as IL-17-expressing T cells (Th17 cells) and follicular T helper cells (Tfh cells) are closely related to many autoimmune pathological conditions. mRNAs encoding Th cell-related cytokines, co-stimulatory molecules or transcriptional factors are dynamically regulated by RBPs for a delicate control of T cell-mediated autoimmune pathology.

#### mRNA degradation

RBPs target multiple Th17 cell-related mRNAs such as STAT3, OX40, IL-17 and granulocyte-macrophage colony-stimulating factor (GM-CSF), and contribute to fine-tuning of Th17-dependent autoimmunity and inflammation. For example, TTP directly binds to the IL-17 mRNA 3′ UTR to mediate mRNA decay.^179^ T cell-specific deficiency of TTP in mice results in spontaneous chronic skin inflammation and severe colitis with an increased Th17 responses and IL-17 production.^180^ In addition, Regnase-1 negatively regulates mRNAs of c-Rel, OX40, and IL-2 through cleavage of their 3′ UTRs in T cells.^181,182^ T cell receptor ligation relieves Regnase-1-mediated suppression of these Th17 genes via cleavage of Regnase-1 at R111 by paracaspase mucosa-associated lymphoid tissue (MALT). Consequently, mice with T cell-specific Regnase-1 deficiency develop spontaneous autoimmune disorders similarly to mice with systemic Regnase-1 deficiency. T cells lacking Roquin also cause inflammatory pathology with increased Th17 cell activation in the lung. Mechanistically, Roquin cooperates with Regnase-1 to repress mRNAs encoding Th17 cell-promoting factors IL-6, inducible T cell costimulator (ICOS), c-Rel, and IRF4, and inhibits Th17 cell differentiation.^183^

The Tfh cells express high levels of CXC chemokine receptor type 5 (CXCR5) and ICOS, secret large amounts of IL-21, and are essential for B cell antibody response and germinal cell (GC) response. Increasing evidence has demonstrated fundamental roles for Tfh cells in autoimmune pathogenesis.^184,185^ Targeting ICOS/ICOS-L interactions or IL-21 pathways shows protective effects in lupus mice.^186^ In the sanroque mouse, Roquin mutation in the ROQ domain causes the development of severe autoimmune lupus-like syndrome mediated by excessive Tfh cell function and GC responses.^187,188^ Mechanistically, Roquin-1 and Roquin-2 post-transcriptionally repress the expression of ICOS and OX40 mRNAs to prevent inappropriate Tfh cell differentiation.^189–191^ Disrupting the interaction between Roquin and Regnase-1 increases Tfh cell differentiation, GC B cell activation and autoantibody formation, indicating the physical interaction of Roquin-1 with Regnase-1 as a brake of autoimmunity.^192^

#### mRNA stabilization

HuR, as an mRNA transcription stabilizer, increases IL-17 and GM-CSF mRNA levels via directly binding to their 3′ UTR, thus promoting Th17 differentiation and pathogenesis during EAE development.^193,194^ HuR also stabilizes mRNA of IRF4 and Runx1, and subsequently promotes RORγt expression and facilitates Th17 cell differentiation and migration into central nervous system. Targeting HuR by its inhibitor DHTS shows effectiveness in delaying the onset and severity of EAE, suggesting HuR as a potential drug target for treating autoimmune neuroinflammation.^195^

Arid5a can stabilize OX40, STAT3, and T-bet mRNAs in T cells to promote Th17- and Th1-dependent pathology in mouse models of EAE.^196–198^ However, Arid5a is shown to be induced by IL-6 in RA patients with the potential function to attenuate Th17 cell differentiation through physically interacting with RORγt.^199^ Mechanisms underlying this discrepancy and the detailed role of Arid5a in competition or cooperation with other RBPs in the development of Th17-dependent autoimmunity remain to be identified.

### RBPs regulate the generation and function of Treg cells in autoimmunity and autoinflammation

Defects of the generation and function of Treg cells have been implicated in various autoimmune disorders.^200^ Changes in the number and function of Treg cells differ among distinct AIDs, indicating that Treg cell-dependent immune tolerance may be disrupted in a context-dependent manner during the development of autoimmunity.

#### mRNA degradation

A subset of CD4^+^ forkhead box protein 3 (Foxp3)^+^Helios^+^ Treg cells are increased in active SLE and positively correlated with the disease’s activity.^201,202^ Compared with Foxp3^+^Helios^–^ Treg cells, Foxp3^+^Helios^+^ Treg cells have stronger suppressive potential with limited expression of IL-2 and IFN-γ. Helios is indispensable for the immune regulatory function of Treg cells as deficiency of Helios causes the spontaneous development of autoimmune pathologies with multi-organ inflammation, systemic inflammation and autoantibody generation.^203,204^ It is recently shown that Zinc finger protein 36 like 2 (ZFP36L2) decreases the expression of Helios (encoded by *Ikzf2*) in Foxp3^+^ Tregs via directly binding to the 3′ UTR of its mRNA and destabilizing the mRNA, leading to the suppression of the iTreg function.^205^ Interestingly, ZFP36L2 also recognizes ARE of the IFN-γ mRNA and blocks its translation in memory T cells, which prevents undesirable protein production from pre-formed cytokine mRNAs under steady state.^206^ Therefore, ZFP36L2 plays variable roles in orchestrating T cell immunity via mRNA destabilization or translational inhibition.

#### m^6^A methylation

m^6^A mRNA methylation is essential for Treg suppressive functions. Treg cell-specific Mettl3-deficient mice exhibit development of autoimmune pathology and loss of Treg suppressive function.^207^ The elevated SOCS caused by Mettl3 deficiency targets the IL-2-STAT5 signaling pathway, eventually resulting in the loss of Treg cell functions. Consistently, m^6^A methyltransferase WT1-associated protein (WTAP) is required for gut RORγt^+^ Treg cell function to prevent colitis as well as for T cell activation and survival. T cell conditional genetic inactivation of WTAP relieves Orai1 and Ripk1 mRNA transcription correlated with T cell activity.^208^ METTL14 deficiency in T cells also causes spontaneous colitis in mice characterized by increased Th1/Th17 response and impaired Treg cell induction, indicating a role for METTL14 in maintaining Treg cells for protection against colitis.^209^

While the impact of m^6^A on Treg cell differentiation has been evidentially reported, their roles in effector T cell differentiation remain elusive. METTL3 is essential for T cell homeostasis and differentiation as depletion of m^6^A in CD4^+^ naïve T cells increases SOCS mRNA and thereby inhibits IL-7-STAT5 signaling and T cell homeostatic proliferation and differentiation.^210^ METTL3 also promotes Tfh cell differentiation and GC response by favoring Tfh transcriptional program and stabilizing Tcf7 transcripts via m^6^A modification.^211^ However, METTL3/METTL14 are also shown to catalyze m^6^A modification on ICOS transcripts to reduce ICOS expression and thus attenuate Tfh cell differentiation.^212^ Therefore, m^6^A modification targets various mRNAs in different T cell subsets, leading to complex regulatory effects in controlling the differentiation, activation and function of T cells.

#### ncRNA regulation

Follicular regulatory T cells (Tfr cells) are differentiated from Treg cells and directly inhibit Tfh and B cell responses in GC. Similar to Tfh cells, Tfr cells express high levels of B-cell lymphoma 6 (Bcl-6) and CXCR5. However, Tfr cells also express Treg cell markers including Foxp3, CD25 and CTLA-4. The generation of Tfr cells depends on transcriptional factors Bcl-6 and NFAT2 and is regulated in an mTORC1 signaling-dependent manner.^213–215^ Roquin upregulates PTEN expression through antagonizing miR-17–92 binding to an overlapping *cis*-element in the 3′ UTR of PTEN mRNA. PTEN upregulation by Roquin inhibits mTOR signaling and suppresses the conversion of Treg to Tfr cells.^216^ However, it remains unclear how altering the conversion of non-follicular Treg cells to Tfr cells eventually affects the final outcome of autoimmunity, given their respective indispensable role in limiting effector T cells and Tfh cells.

We recently show that T cell expression of lncRNA-GM promotes T cell-mediated autoimmunity via polarizing Th17 differentiation but inhibiting iTreg differentiation, in a manner dependent on its ability of binding to transcription factor Foxo1 and reducing Foxo1 activity.^217^ lncRNA-GM is also expressed in macrophages, while viral infection can downregulate lncRNA-GM to facilitate viral escape by inhibiting the kinase activity of TBK1.^218^ Therefore, ncRNAs can interact with distinct protein partners in a cell type-specific manner. Further elucidation of the expression and function of ncRNA–RBP complexes in the development of tissue inflammation and AIDs will provide potential therapeutic strategies to treat autoimmune inflammation.

### RBPs regulate B cell activation and GC response in autoimmunity and autoinflammation

Abnormal B cell activation and breakdown of B cell tolerance are critical for the excessive autoantibody production leading to autoimmunity.^219^ A unique subset of regulatory B cells mediate suppression of autoimmunity and inflammation via producing anti-inflammatory cytokines IL-10 and TGF-β and shaping the inhibitory immune microenvironment.^220^ Regulation of B cell differentiation, activation and antibody production via post-transcriptional mechanism has attracted increasing attention.

#### mRNA degradation

RBPs controlling mRNA degradation and stability are essential for regulating B cell activation and responses in autoimmunity. ZPF36L1 promotes marginal zone B cell localization and survival, partly through its ability in limiting the expression of transcription factors kruppel like factor 2 (KLF2) and IRF8 post-transcriptionally.^221^ Regnase-1 maintains B cell homeostasis by suppressing the BCR-driven transcriptome changes; however, its specific mRNA targets remain unclear.^222^ RBP, such as ZFP36 family members, Regnase-1 has broad and distinct roles in fine-tuning the overactivation of innate and adaptive immune responses. It therefore will be important to further investigate how RBPs coordinate these cell type-dependent post-transcriptional regulations of mRNA stability and translation in distinct immune responses.

#### Alternative splicing

Mature B cells undergo alternative splicing for the expression of IgM/IgD from primary RNA transcripts, a process which determines the B cell antigenic specificity in discrimination between self and non-self antigens. Zinc-finger protein 318 (ZFP318) is essential for IgD expression mainly via modulating alternative mRNA splicing.^223^ The key homologous recombination factor, radiation-sensitive 52 (Rad52) mediates IgD class-switch DNA recombination in concert with ZFP318 downregulation, and Rad52 phosphorylation is associated with high levels of IgD autoantibodies in SLE patients and in lupus mice.^224^

Alternative splicing is also important for regulation of mRNAs related to BCR signaling or functions. For example, HuR promotes B cell differentiation and activation through modulating B cell metabolism via mRNA splicing. B cell-specific ablation of HuR results in defective mitochondrial metabolism and accumulation of reactive oxygen species, leading to impaired B cell survival and proliferation. Mechanistically, HuR modulates splicing of dihydrolipoamide S-succinyltransferase (Dlst) mRNA, thus enhancing Dlst translation into a subunit of the 2-oxoglutarate dehydrogenase complex (αKGDH) with enzymatic activity. This study describes how RBP-mediated post-transcriptional regulation modulates metabolic switch during B cell activation and differentiation.^225,226^ Another RBP, polypyrimidine tract binding protein 1 (PTBP1) promotes B cell proliferation and activation in GC via controlling alternative splicing of transcripts of c-Myc target genes such as *Pkm* and *Tyms*.^227^ As PTBP1 is involved in many post-transcriptional regulations such as alternative splicing, alternative polyadenylation, mRNA decay and translational regulation,^228,229^ it is important to investigate how these distinct regulatory mechanisms contribute to PTBP1-mediated control of B cell responses and how these biological processes eventually affect the outcome of autoimmune response.

#### ncRNA regulation

ncRNAs play important roles in B cell-dependent autoimmunity and may serve as potential targets for treatment of AIDs. For example, miR-7, miR-21 and miR-22 are highly expressed in B cells and play an important function in increasing BCR signaling by suppressing the expression of PTEN, and thus contribute to B cell hyperactivity and autoantibody production in SLE. Antagonizing miR-7 can suppress B cell hyperactivity, Tfh expansion and GC response, and consequently reduce the lupus manifestations in MRL^lpr/lpr^ lupus mice, indicating miR-7 as a potential therapeutic target of SLE.^230,231^ It will be interesting to clarify whether potential RBPs are involved in the upregulation of miR-7 to prevent its expression or function at steady state for the maintenance of B cell tolerance.

In addition, lncRNAs have been shown to regulate B cell development and activation, and lncRNA dysregulations are associated with various autoimmune pathologies. Abnormal X-chromosome inactivation (XCI), reflected by reduced XIST RNA and H2AK119Ub enrichment at the inactive X chromosome is present in B cells of both pediatric and adult SLE patients.^232^ Functionally, XIST enforces X-inactivation in adult human B cells via binding to tripartite motif containing 28 (TRIM28) that mediates Pol II pausing at promoters of X-linked genes such as *TLR7* in B cells. XIST loss and TLR7 stimulation promote CD11c^+^ atypical B cell formation which is implicated in SLE pathogenesis.^233^ Consistently, a gain-of-function variant of TLR7 (Y264H) has been recently identified to drive aberrant B cell activation and CD11c^+^ age-associated B cell accumulation, and cause SLE in humans, highlighting the importance of TLR7 expression and function for human autoimmunity.^234^ Further investigations will be necessary to elucidate how the XIST-interacting RBPs such as spen family transcription repressor (SPEN), TRIM28 and PTBP1 contribute to the disruption of XIST-dependent XCI maintenance and development of female-biased autoimmunity such as SLE.

#### Anti-RBP antibodies

RBPs can also be recognized as autoantigens, and anti-RBP antibodies are frequently detected in patients of AIDs such as SLE.^235^ For example, anti-Sm antibodies that interact with U1, U2, U4 and U5 RNA snRNPs are predictive for SLE classification; anti-SS-A (Ro52/Ro60) autoantibodies are described as serological markers for Sjögren’s syndrome but also detected in other AIDs. Autoantibodies against hnRNP-A2 and hnRNP-A2-specific T cells are detected in patients with SLE.^96^ Anti-RBP antibodies are predominantly produced by long-lived plasma cells and maintained at a relatively stable level over time. These antibodies recognize RNA–protein complexes and form immune complexes (ICs) that induce autoimmune activation. While the formation and tissue deposition of DNA ICs lead to tissue inflammation, cytokine production and complement activation, the RNA ICs are also implicated in IFN production and plasma cell overactivation.^236^ It will be important to systemically clarify the signatures of expression of anti-DNA or anti-RBP antibodies in various stages or subtypes of AIDs and how the distinct expression patterns of anti-RBP antibodies could affect disease progression or therapy responsiveness.

## Conclusions and perspectives

Despite substantial achievements in revealing the role of RBPs and RNA metabolism in the autoimmune response and inflammatory pathogenesis, many important questions in this field still remain unclear and require future investigations.

1. RBP–RNA interactions are dynamically remodeled in different autoimmune and inflammatory contexts. Large-scale analysis of RNA-binding proteome and RBP interactome will facilitate the identification of novel canonical and non-canonical RBPs and their dynamic interactions with RNA or proteins implicated in the regulation of autoimmunity and autoinflammation.
2. RBPs can modulate many cellular events including gene transcription, RNA regulation, protein modification and function during autoimmunity. The molecular basis for the RBP-centered molecular machineries consisting of DNAs, RNAs and proteins, e.g., chromatin-binding RBP complexes and post-translational modification networks of RBPs, in the regulation of autoimmunity remains to be revealed.
3. RNA stability and metabolism are critical for both activation and suppression of inflammation. Further investigations of the role of RBP–RNA interactions in the inflammation resolution programs will provide new opportunities for the development of therapeutic approaches that selectively facilitate inflammation resolution while maintaining immune defense and tissue homeostasis.
4. Metabolic abnormities in various innate and adaptive immune cells are closely associated with autoimmune pathogenesis. How RBPs and RNA functions affect the key metabolic events such as nutrient uptake, energy homeostasis, and glucose, amino acid and lipid metabolism in different immune cells to control autoimmunity and autoinflammation remains to be identified.
5. How RBP–RNA networks influence the differentiation and function of specialized immune and non-immune cell subsets at single-cell level remains largely unknown. Integrative analysis of multi-omics data will deepen our understanding of spatiotemporal interplay between genetic, epigenetic and signaling networks and single-cell plasticity during autoimmunity and autoinflammation.
6. Mechanisms that regulate RBP trafficking between the nucleus and the cytoplasm during immune responses remain unclear. The application of high-resolution in situ molecular imaging techniques will enable real-time visualization of communications between RBPs and RNAs or DNAs in different cellular compartments within live tissues and contribute to a better understanding of dynamic spatiotemporal responses of RBPs in autoimmune processes.
7. The signatures and functions of RNA modification and higher-order structures in autoimmunity remain poorly understood. The characterization of novel chemical modifications in RNA sequences through mass spectrometry-based technologies and identification of RBPs that install, recognize, and remove these modifications will shed new light on the structural and functional roles of dynamic RBP–RNA networks in autoimmunity and suggest potential drug targets.
8. A crucial role of the complement system in metabolic remodeling and tissue inflammation and their potential as drug targets for relieving tissue damage of AIDs are increasingly shown. Understanding how the complement system and the related coagulation pathways are regulated by RBP–RNA interactions will provide clues for targeting related pathways in diseases.
9. The exact role and mechanisms of RBP dysregulations and RNA functional abnormalities in autoimmunity and autoinflammation have been demonstrated mostly in animal models. Large cohort studies and clinical translational investigations are required to investigate how RBP–RNA regulatory networks are functionally linked with immunological phenotype and clinical manifestations in human autoimmunity.

In sum, RBPs play critical roles in the regulation of autoimmunity and autoinflammation at different steps and via different mechanisms. Proinflammatory RBPs promote innate inflammatory responses and GC responses via strengthening inflammatory mRNA expression and translation or constraining inhibitory immune signaling. On the other hand, a larger number of anti-inflammatory RBPs suppress innate inflammatory responses and adaptive immunity by post-transcriptional or translational mechanisms, and consequently serve as essential safeguards against the development of harmful AIDs. These proinflammatory and anti-inflammatory RBPs precisely regulate the formation and function of organ inflammation niche and determine the initiation, activation, and resolution of distinct autoimmune inflammatory responses. The imbalance of proinflammatory and anti-inflammatory RBPs results in exacerbation and progression of organ pathophysiology by organizing inflammation niches that drive sustained inflammatory cell infiltration and tissue injury.

Numerous drug candidates targeting key signaling molecules of the innate or adaptive immunity for treating AIDs are being evaluated in preclinical or clinical studies. Some of these drug candidates have achieved exciting results while others are disappointing due to unfavorable side effects such as infections. Cell type-selective and context-dependent functions of RBPs in maintaining the immune tolerance have made RBPs promising targets for precise therapeutics that target only the pathogenic autoimmune pathways without affecting protective immune defense. For example, enhancing TTP mRNA stability through genetically deleting a 136-base instability motif in the 3′ UTR of TTP mRNA has been shown to protect mice against autoimmune pathogenies and reduce the severity of collagen antibody-induced arthritis, imiquimod-induced dermatitis or EAE.^237^ In vivo treatment of PP2A agonists which can dephosphorylates and thereby activates TTP, ameliorates the autoimmune phenotype of inflammatory arthritis in mice.^238^ These findings suggest forced expression or activation of anti-inflammatory RBPs such as TTP as a promising therapeutic strategy against AIDs. Further investigations of how the RBP-mediated regulatory networks consisting of DNA, RNAs and proteins are associated with disease susceptibility and development of systemic or organ-specific inflammatory pathologies will contribute to a better understanding of autoimmune pathogenesis and lead to therapeutic breakthroughs.
